# A systems biology approach to predict and characterize human gut microbial metabolites in colorectal cancer

**DOI:** 10.1038/s41598-018-24315-0

**Published:** 2018-04-18

**Authors:** QuanQiu Wang, Li Li, Rong Xu

**Affiliations:** 1 ThinTek LLC, Palo Alto, California, 94306 USA; 2 Department of Family Medicine and Community Health, Case Comprehensive Cancer Center, School of Medicine, Case Western Reserve University, Cleveland, OH USA; 3 Department of Population and Quantitative Health Sciences, School of Medicine, Case Western Reserve University, 2103 Cornell Road, Cleveland, Ohio, 44106 USA

## Abstract

Colorectal cancer (CRC) is the second leading cause of cancer-related deaths. It is estimated that about half the cases of CRC occurring today are preventable. Recent studies showed that human gut microbiota and their collective metabolic outputs play important roles in CRC. However, the mechanisms by which human gut microbial metabolites interact with host genetics in contributing CRC remain largely unknown. We hypothesize that computational approaches that integrate and analyze vast amounts of publicly available biomedical data have great potential in better understanding how human gut microbial metabolites are mechanistically involved in CRC. Leveraging vast amount of publicly available data, we developed a computational algorithm to predict human gut microbial metabolites for CRC. We validated the prediction algorithm by showing that previously known CRC-associated gut microbial metabolites ranked highly (mean ranking: top 10.52%; median ranking: 6.29%; p-value: 3.85E-16). Moreover, we identified new gut microbial metabolites likely associated with CRC. Through computational analysis, we propose potential roles for tartaric acid, the top one ranked metabolite, in CRC etiology. In summary, our data-driven computation-based study generated a large amount of associations that could serve as a starting point for further experiments to refute or validate these microbial metabolite associations in CRC cancer.

## Introduction

Colorectal cancers are the second leading cause of cancer-related deaths in in the United States and the third most common cancer in men and in women^1^. In the United States alone, an estimated 135,430 men and women will be diagnosed with CRC in the year 2017 and 50,260 will die from this disease^2^. It is estimated that forty-five percent of CRC are preventable by modifiable environmental factors such as food, nutrition, lifestyle, and physical activity, among others^3,4^.

Human gut microbiota, the collection of microorganisms that live in the human digestive tracts, play central roles in human health and diseases, by metabolizing nutrients and food components and by controlling the immune response of the human body^5–8^. Growing evidence suggests that gut microbiota and their metabolites not only influence carcinogenesis and tumor progression, but also influence the efficacy of anticancer therapies^9–11^. Human microbiome (the collective genomes of the microbiota) studies have revealed that gut dysbiosis (an imbalance in the intestinal bacteria) is associated with the increased incidence of CRC^11–14^.

Undigested dietary components that reach the large intestine are fermented by microbiota to produce a variety of metabolites and nutrients. It has become increasingly clear that the collective metabolic outputs of gut microbiota strongly influence cancer susceptibility and progression^5,15,16^. For example, recent studies have shown that the short-chain fatty acid (SCFA) butyrate, one of the most abundant metabolites of gut microbiota in the fermentation of fiber, has a role in the suppression of inflammation and colorectal cancer^17^.

Currently, the mechanisms by which gut microbial metabolites interact with host genetics in promoting or protecting against CRC remain unknown. Computational approaches have been widely used in drug development^18–24^ and disease mechanism understanding^25–27^. We have recently developed a hypothesis-driven systems approach to understand how trimethylamine N-oxide (TMAO), a gut microbial metabolite of dietary meat and fat, is mechanistically involved in CRC^28^. Here, we present a data-driven computational approach to estimate which microbial metabolites might affect CRC. The identification of human gut microbial metabolites and the understanding of their role as key mediators through which bacteria might promote or protect against CRC is important. Taken together, characterizing these microbial metabolites will likely enhance our understanding of the complex gene-environment interactions in carcinogenesis, and add up to new possibilities for CRC diagnosis, prevention, and treatment.

## Data and Methods

### Data

**Figure 1:**
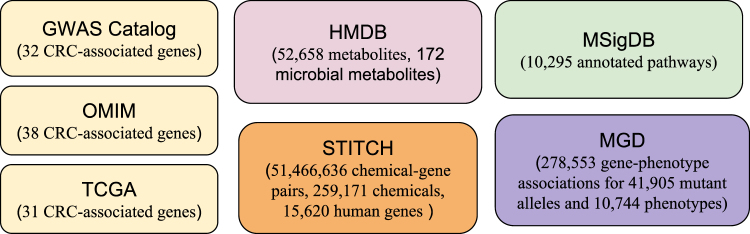
Datasets used in this study.

We used large amounts of publicly available data, including human metabolome, disease genetics, chemical genetics, signaling pathways, and mouse genome-wide mutation phenotypes for both the prediction and functional characterization of gut microbial metabolites for CRC (Fig. 1).

#### Data resources of CRC-associated genes

We used three complementary disease genetics resources to obtain CRC-associated genes. We obtained (1) 32 CRC-associated genes from the Catalog of Published Genome-Wide Association Studies (GWAS), a comprehensive collection of all published GWAS studies^29^; (2) 38 CRC-associated genes from the Online Mendelian Inheritance in Man (OMIM), a comprehensive collection of human genes and genetic phenotypes for Mendelian disorders^30^; and (3) 31 genes that are significantly mutated in colorectal cancer patients from the Cancer Genome Atlas (TCGA), a comprehensive cancer database and contains genetic and clinical data for 283 colorectal patients^31^. We used these three complementary and independent disease genetics resources to demonstrate the robustness of the algorithms and our findings.

#### The Human Metabolome Database (HMDB)

HMDB is a comprehensive database of small molecule metabolites found in the human body^32^. Currently, HMDB contains 52,658 metabolites, including 172 metabolites originated in human gut microbiota.

#### Data resource of metabolite-associated genes

We obtained metabolite/chemical-associated genes from STITCH (Search Tool for Interactions of Chemicals). STITCH contains chemical-gene association data for > 300,000 small molecules and 2.6 million proteins from 1,133 organisms^33^. We used chemical-gene associations found in human body, which include 1,466,636 chemical-gene pairs, 259,171 chemicals, and 15,620 human genes.

#### Genetic pathway data

We used gene-pathway association data from the Molecular Signatures Database (MSigDB) to construct molecular profiles for CRC and metabolites and to study the interplaying pathways underlying top identified microbial metabolites and CRC. MSigDB contains 10,295 annotated pathways and gene sets^34^.

#### Genome-wide mutational phenotypes in experimental mouse models

Recently, the Mouse Genome Database (MGD) has made available large amounts of phenotypic descriptions of systematic gene knockouts in mouse models^35^. We have recently shown that these strong causal gene-phenotype annotations (278,553 gene-phenotype associations for 41,905 mutant alleles and 10,744 phenotypes) have great potential for virtual phenotypic screening for drug discovery^21–23^. In this study, we used gene-phenotype associations from MGD to assess the functional effects of top ranked microbial metabolites on CRC-related phenotypes.

### Metabolome-wide prediction of gut microbial metabolites for CRC

**Figure 2:**
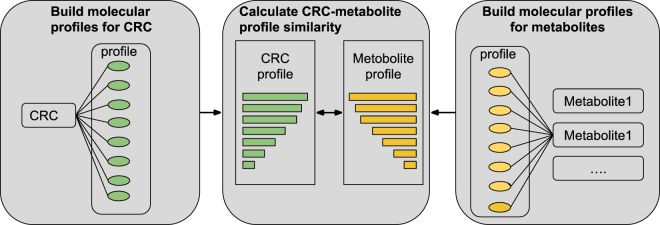
Rank metabolites for CRC based on profile similarities.

The experimental flowchart is summarized in Fig. 2 and described in details in subsequent sections.

#### Construct molecular profiles for diseases

We identified CRC-associated genes from the three disease genetics databases: the GWAS catalog, OMIM, and TCGA. Pathways associated with each gene were obtained from MSigDB. For each pathway, we assessed its probability of being associated with the given set of CRC-associated genes as compared to its probability associated with the same number of randomly selected genes. The random process is repeated 1000 times and a t-test was used to assess the statistical significance. As an example, the pathway “*colorectal cancer*” is associated with 7 out of 31 (29.0%) CRC genes from TCGA, which represents a significant 39-fold enrichment as compared to the random expectation of 0.7%. The molecular profile for CRC consists of a list of significantly enriched pathways. Three molecular profiles were built for CRC using genes from three complementary disease genetics resources.

#### Build molecular profiles for chemicals/metabolites

Similarly, we built one molecular profile for each of the 259,170 chemicals/metabolites from the STITCH database. For example, the molecular profile for butyric acid, a human gut microbial metabolite, consists of 609 pathways.

#### Prioritize metabolites for CRC

Metabolites were prioritized based on how their molecular profiles are similar to CRC-specific molecular profile. We implemented three commonly used set similarity measures: overlap, Jaccard coefficient, and cosine similarity^36^. Overlap is defined as the intersection of disease profile set and metabolite profile set. Jaccard coefficient of two sets is defined as the size of the intersection divided by the size of the union. The cosine similarity is defined as the Euclidean dot product of two sets. The output is a ranked list of 259,170 chemicals/metabolites for CRC prioritized based on their profile similarities with CRC.

#### Evaluation using known CRC-associated metabolites

We evaluated the prioritization algorithm using 32 known CRC-associated metabolites extracted from a recent review paper^15^. Recall, mean and median rankings were used for performance measures. Significance was calculated by comparing actual rankings to random expectation (based on random expectation, a metabolite shall have an average ranking of 50%). We also examined the number of known metabolites at 10 decile rankings. A good prioritization algorithm shall enrich true positives among top-ranked entities. We calculated the number of known metabolites at each decile and plotted decile enrichment distribution.

#### Evaluate the rankings of all human microbial metabolites for CRC

We investigated whether human gut microbial metabolites in general are highly related to CRC in terms of molecular relevance. We examined the rankings of all 172 microbial metabolites among prioritized chemicals. Recall, mean and median rankings were calculated and decile ranking was plotted.

### Functional characterization of top ranked novel microbial metabolite in CRC

#### Identify common pathways between novel metabolite and CRC

We identified common genetic pathways that are significantly enriched for the novel metabolite and CRC. We then developed an algorithm to further prioritize these common pathways. The ranking of each common pathway is a balance measure of rankings from the disease (CRC) and from the metabolite. A pathway ranks highly if and only if it ranks highly for both the metabolite and the disease. The ranking of a common pathway is defined as: *ranking_combined* = 2*(ranking_d * ranking_m)/(*ranking_d* + *ranking_m*), where *ranking_d* is the ranking score of a pathway for CRC; and *ranking_m* is the ranking score of the same pathway for the metabolite.

#### Functional characterization of phenotypic effects of the novel metabolite on CRC

We obtained metabolite-associated genes from STITCH and then mapped genes to their corresponding mouse gene homologs (e.g., SMAD4 =  > 18Wsu70e) using human-mouse homolog mapping data from MGD^35^. The mapped mouse genes were then linked to their corresponding mutational phenotypes in mouse models (e.g., SMAD4 =  > *increased intestinal adenoma incidence*, TP53 =  > *colon polyps*) using gene-phenotype association data from MGD. For each mapped phenotype, we assessed its probability of being associated with the given set of metabolite-associated genes as compared to its probability associated with the same number of randomly selected genes. The random process is repeated 1000 times and a t-test was used to assess the statistical significance. Similarly, we built a CRC-specific phenotype profile using data from disease genetics databases. CRC- and metabolite-specific phenotype profiles were intersected. Shared phenotypes were prioritized as described for prioritizing shared pathways between the metabolite and CRC. A phenotype (e.g., *colon polyps*) ranked highly if and only if it ranks high for both CRC and the novel metabolite.

### Data availability

http://nlp.case.edu/public/data/CRC_Microbiome/.

## Results

### Known CRC-associated microbial metabolites ranked highly

The algorithm found 26 of 32 known metabolites (recall: 0.813) and the recall is determined by the coverage of the STITCH database. The not-perfect recall indicates that although STITCH is currently the most comprehensive chemical genetics database of 1,466,636 chemical-gene pairs for 259,171 chemicals/metabolites, the coverage for gut microbial metabolites is not perfect. Known CRC metabolites ranked significantly high as compared to random expectation (Table 1). As an example, when CRC-associated genes from the GWAS catalog were used to build disease-specific molecular profile, known CRC-associated microbial metabolites on average ranked at top 10.52% among 259,170 chemicals/metabolites, which is significantly higher than random expectation (P-value: 3.85E-6). These findings are consistent when CRC-associated genes from three complementary disease genetics data resources were used (Table 1).

**Table 1:** Known CRC-associated microbial metabolites were ranked highly among 259,170 chemicals/metabolites when three complementary disease genetics databases (The GWAS Catalog, OMIM and TCGA) were used for obtaining CRC-associated genes.

Disease Genetics	Recall	Mean Ranking (top %)	Median ranking (top %)	P-value
GWAS	0.813	10.52%	6.29%	3.85E-16
OMIM	0.813	12.67%	9.51%	1.62E-14
TCGA	0.813	12.21%	11.60%	9.38E-15

**Figure 3:**
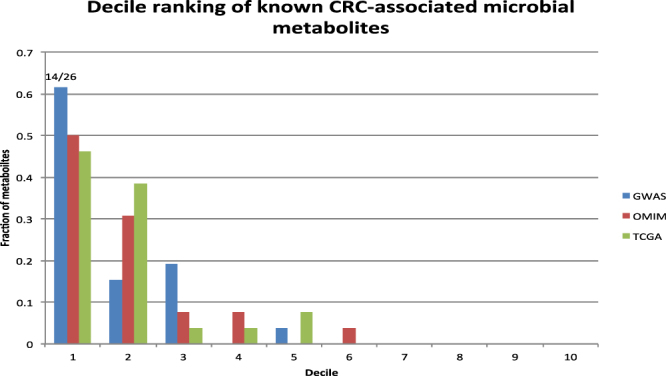
The decile ranking of known CRC-associated metabolites among 259,170 chemicals/metabolites. Three complementary disease genetics databases (The GWAS Catalog, OMIM and TCGA) were used for obtaining CRC-associated genes.

The decile rankings further demonstrate that the ranking algorithm effectively enriched known CRC-associated metabolites at top. For example, 14 of 26 known metabolites are ranked at the first decile (top 10%) (Fig. 3).

We then examined which categories of microbial metabolites ranked highly for CRC. We classified known metabolites into six categories^15^. Short chain fatty acids (SCFAs) are known to be involved in colon health^15,17^. Our study indeed shows that SCFAs ranked highest (top 3.86%) among all known CRC-associated metabolites. The results are consistent when three independent disease genetics databases were used to obtain CRC-associated genes (Table 2).

**Table 2:** Stratified rankings of known CRC-associated microbial metabolites among 259,170 prioritized chemicals/metabolites. Three complementary disease genetics databases (The GWAS Catalog, OMIM and TCGA) were used for obtaining CRC-associated genes.

Metabolite	Disease Genetics	Recall	Mean Ranking (top %)	Median ranking (top %)	P-value
SCFAs	GWAS	1.00	3.86%	4.65%	3.07E-6
	OMIM	1.00	5.67%	5.53%	1.62E-5
	TCGA	1.00	6.84%	5.53%	5.29E-5
Bile acids	GWAS	0.78	7.06%	1.56%	1.60E-5
	OMIM	0.78	6.34%	2.91%	3.67E-6
	TCGA	0.78	6.20%	3.10%	3.22E-6
Indoles	GWAS	0.60	9.51%	8.76%	0.005
	OMIM	0.60	10.98%	11.24%	0.011
	TCGA	0.60	10.21%	13.05%	0.005
Cresols	GWAS	0.75	11.11%	10.51%	0.003
	OMIM	0.75	16.29%	14.04%	0.006
	TCGA	0.75	14.93%	14.50%	0.002
Phenolic acids	GWAS	0.80	18.34%	20.42%	0.010
	OMIM	0.80	20.43%	21.16%	0.019
	TCGA	0.80	20.77%	17.93%	0.027
Polyamines	GWAS	1.00	23.12%	28.46%	0.149
	OMIM	1.00	31.03%	38.81%	0.328
	TCGA	1.00	27.11%	34.39%	0.204

### Human gut microbial metabolites in general ranked highly for CRC

Microbial metabolites in general are highly associated with CRC based on molecular convergence. The algorithm found 131 of 172 (recall: 0.761) metabolites originated in gut microbiota (as determined by HMDB database)^32^. These 131 microbial metabolites ranked consistently highly when CRC-associated genes from three complementary databases were used (Table 3). As an example, microbial metabolites on average ranked at top 14.43% (P-value: 2.27E-57) among 259,170 prioritized chemicals/metabolites.

**Table 3:** Rankings of gut microbial metabolites among 259,170 prioritized chemicals/metabolites.

Disease Genetics	Recall	Mean Ranking (top %)	Median ranking (top %)	P-value
GWAS	0.761	14.43%	10.11%	2.27E-57
OMIM	0.761	16.88%	12.13%	2.77E-46
TCGA	0.761	18.84%	12.47%	2.47E-37

**Figure 4:**
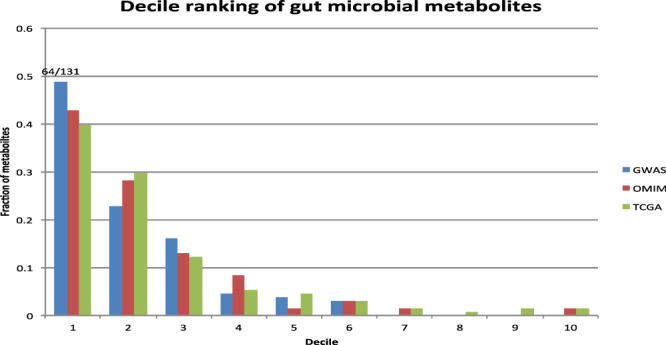
The decile ranking of all human gut microbial metabolites among 259,170 chemicals/metabolites. Three complementary disease genetics databases (The GWAS Catalog, OMIM and TCGA) were used for obtaining CRC-associated genes.

The decile rankings show that the majority of gut microbial metabolites were ranked at first decile (top 10%) (Fig. 4). For example, when CRC-associated genes from the GWAS catalog were used to build disease-specific molecular profile, 64 of 131 gut microbial metabolites were ranked at the first decile.

The top 20 ranked microbial metabolites are shown in Table 4. Seven of these top 20 metabolites are known CRC-associated metabolites. Tartaric acid ranked at top 3, immediately following butyric acid, a well-known microbial metabolite associated with CRC and colon health. Trimethylamine n-oxide (TMAO) also ranked highly (top 13). Previous studies showed that TMAO is both mechanistically and clinically associated with increased risk of CRC^28,37^.

**Table 4:** Top 20 ranked microbial metabolites for CRC. Seven known CRC-related microbial metabolites are highlighted in green.

Rank	Metabolite	Rank	Metabolite
1	Taurochenodesoxycholic acid	11	Isopropyl alcohol
2	Butyric acid	12	D-alanine
3	Tartaric acid	13	Trimethylamine n-oxide
4	Acetaldehyde	14	Taurodeoxycholic acid
5	Mannitol	15	Deoxycholic acid glycine conjugate
6	P-aminobenzoic acid	16	Acetone
7	Trans-ferulic acid	17	Zeaxanthin
8	Putrescine	18	3,4-dihydroxybenzeneacetic acid
9	Chenodeoxycholic acid glycine conjugate	9	1-butanol
10	D-glutamic acid	20	Phenylethylamine

### Tartaric acid may be both genetically and functionally involved in CRC

Tartaric acid is the top one ranked microbial metabolite that is not included in the list of 32 known CRC-associated metabolites. Tartaric acid is a phytochemical found abundantly in nuts, apricots, apples, sunflower, avocado, grapes, among others^38^. Tartaric acid is associated with 305 genes (based on the STITCH database). These genes are significantly associated with 611 pathways, demonstrating that tartaric acid may participate in many biological functions. Many of the top ranked pathways are related to immune functions, including *Cytokines and Inflammatory Response*, *IL12-mediated signaling events*, and *Downstream signaling in naïve CD8* + *T cells*. Among 117 pathways significantly associated for CRC, 64 (54.7%) pathways are also associated with tartaric acid, demonstrating that tartaric acid may be mechanistically involved in CRC etiology. The top 20 pathways shared by both CRC and tartaric acid are shown in Table 5. Many of these top common pathways are directly involved in CRC, including *Regulation of nuclear SMAD2/3 signaling*, *β-catenin pathway*, *WNT pathway*, *TGF-beta signaling pathway*, and *Colorectal cancer*.

**Table 5:** Top 20 pathways significantly associated with both CRC and tartaric acid.

Rank	Common Pathway	Rank	Common Pathway
1	Regulation of nuclear SMAD2/3 signaling	11	Validated targets of C-MYC transcriptional activation
2	Integrin cell surface interactions	12	Regulation of retinoblastoma protein
3	ECM-receptor interaction	13	BMP receptor signaling
4	Small cell lung cancer	14	Prostate cancer
5	Beta-catenin pathway	15	Arrhythmogenic right ventricular cardiomyopathy (ARVC)
6	Progesterone-mediated oocyte maturation	16	Wnt-mediated signal transduction
7	Signaling by SCF-KIT	17	Colorectal cancer
8	Beta1 integrin cell surface interactions	18	ErbB4 signaling events
9	AP-1 transcription factor network	19	Internalization of ErbB1
10	TGF-beta signaling pathway	20	Beta3 integrin cell surface interactions

To estimate the possible effects of tartaric acid on CRC, we identified mouse mutational phenotypes that are significantly associated with both CRC and tartaric acid. A total of 2441 mouse mutational phenotypes are significantly associated with tartaric acid, and 600 phenotypes are significantly associated with CRC. Among the 600 CRC-associated phenotypes, 267 (45%) phenotypes are also associated with tartaric acid. The top 20 shared phenotypes are shown in Table 6. Both top 1 and 2 ranked phenotypes are directly related to digestive system.

**Table 6:** Top 20 phenotypes significantly associated with both CRC and tartaric acid. CRC-specific phenotypes are highlighted (yellow).

Rank	Common Phenotype	Rank	Common Phenotype
1	Abnormal intestinal goblet cell morphology	11	Kidney failure
2	Abnormal intestinal epithelium morphology	12	Increased osteoclast cell number
3	Abnormal forelimb morphology	13	Abnormal metastatic potential
4	Abnormal osteoclast physiology	14	Abnormal renal tubule morphology
5	Albuminuria	15	Abnormal head morphology
6	Abnormal facial morphology	16	Increased bone mineral density
7	Abnormal lymphopoiesis	17	Abnormal vascular wound healing
8	Glomerulosclerosis	18	Increased lymphocyte cell number
9	Decreased susceptibility to injury	19	Abnormal pancreatic islet morphology
10	Increased circulating creatinine level	20	Abnormal hindlimb morphology

## Discussion

More than half the cases of cancer, including CRC, occurring today are preventable and about one-third of the cases can be attributed to modifiable environmental factors such as food, nutrition, lifestyle, and physical activity, among others^4^. The susceptibility, initiation, and progression of CRC and many other cancers is primarily driven by gene–environment interactions. Human gut microbiota are important modifiable environmental factors that are part of the ecosystem of our bodies^.^ Functional studies in germ-free mouse models of cancer have demonstrated that microbiota can affect cancer susceptibility and progression in various organs, including colon, however, the mechanisms by which gut microbial metabolites are involved in cancer remain unknown.

In this study, we presented a data-driven systems approach to identify and estimate gut microbial metabolites playing a role in CRC. Our approach is a data-driven computational estimation, which can be applied to different traits and diseases. In this study, we focused on CRC because of the vast availability of known CRC-associated gut microbial metabolites. Our data-driven computational method to estimate associations can take a disease name or a list of disease-associated genes as input, and the output will be a ranked list of microbial metabolites (along with shared molecular signatures and functional phenotypes) for the input disease. For clarity, this ‘*in silico*’ study is not functional microbiome study. Instead, it largely complements existing microbiome studies by identifying microbial metabolites within vast amounts of existing database information of diseases, genes, pathways, functional phenotypes, and metabolome.

However, our study holds several limitations that warrant further discussion. First, the relationships among microbes, their metabolites, and hosts are complex, non-linear and bi-directional^39^. For example, recent studies showed that gut microbial metabolites are involved in CRC etiology by altering host epigenome^37^. Our study focused on the database-dependent interactions between microbial metabolites and CRC genetics of the host. Indeed, we lack the necessary data in order to computationally model the effects of metabolites on microbe populations, the host genetics on microbe variations or epigenetic effects on host genomes. The goal of this study was to provide estimates of associations between human gut microbial metabolites and CRC, which in turn may inform the identification of responsible microbe composition in cancer etiology.

Second, host genetics can affect gut microbiota composition and metabolic outputs in response to environmental factors^40,41^. A person’s genetic make-up can influence his/her response to environmental stressors, gut microbiota population, and microbiome-genome interactions. As personal genetic and genomics information becomes increasingly available, a patient-focused understanding of environment-microbiome-genome-cancer interactions is possible by linking personal genome to metabolite-gene-pathway-disease connections as identified in this study.

Third, among the 41,806 small molecule metabolites available in HMDB, only 172 metabolites (~0.4%) originate in gut microbiota. The field of microbiome research is a fast-moving target with an increasing number of microbial metabolites being identified. The computational algorithms we developed have built-in flexibility and capability to incorporate new data as it becomes available.

Lastly, our current study is pure ‘*in-silico*’. Our goal was to generate estimates of associations data/hypotheses that may be tested to refute or validate our suggestions of gut microbial metabolites role in CRC. We anticipate that both the data-driven computational methods developed and the associations generated in this study will likely stimulate further studies of microbiome-gene interactions in cancer etiology. Taken together, validating identified metabolite-CRC associations in animal models or humans are needed in order to translate the findings into cancer diagnosis, prevention, and treatment.
